# The Effect of Heat Stress During the Insemination Period on the Conception Outcomes of Dairy Cows

**DOI:** 10.3390/ani15132001

**Published:** 2025-07-07

**Authors:** Wissem Baccouri, George Wanjala, Violetta Tóth, István Komlósi, Edit Mikó

**Affiliations:** 1 Doctoral School of Animal Science, University of Debrecen, 4032 Debrecen, Hungary; baccouri.wissem@agr.unideb.hu (W.B.); komlosi@agr.unideb.hu (I.K.); 2 Institute of Animal Sciences and Wildlife Management, Faculty of Agriculture, University of Szeged, 6800 Hódmezővásárhely, Hungary; wanjala.george@szte.hu (G.W.); toth.violetta@szte.hu (V.T.); 3 Department of Animal Science, Institute of Animal Science, Biotechnology and Natural Conservation, Faculty of Agricultural and Food Sciences and Environmental Management, University of Debrecen, Böszörményi út 138, 4032 Debrecen, Hungary

**Keywords:** heat-stress, Holstein-Friesian, reproduction, heritability

## Abstract

This study examined the impact of heat stress during specific periods before and after insemination to identify the most critical time affecting insemination success in dairy cows. A total of 47,199 insemination records from 6751 Holstein-Friesian cows (2002–2024) were analysed using generalised linear mixed models (GLMMs) with a binomial distribution. The periods assessed were the day of insemination, 21–6 days before (P1), 5–2 days before (P2), 1 day before to 1 day after (P3), 2–7 days after (P4), and 8–30 days after insemination (P5). Mild heat stress significantly reduced insemination success on the day of insemination, with a critical THI threshold of 60. Among all periods, P1 was the most sensitive to heat stress, with even mild stress negatively affecting outcomes. Severe heat stress (THI ≥ 72) also impacted P3 and P5, whereas P2 and P4 showed no significant effect. Heritability estimates increased with heat stress intensity, suggesting genetic variability in resilience. These findings highlight the importance of managing heat stress from three weeks before to one month after insemination and support the potential for genetic selection to improve reproductive performance under heat stress conditions.

## Simple Summary

Heat stress can negatively affect the ability of dairy cows to become pregnant after insemination. This study looked at over 47,000 insemination records from more than 6000 cows to find out which periods around insemination are most sensitive to heat. The objective was to understand when cows are most at risk to enable farmers and researchers to take action to improve fertility. This study focused on different time frames before and after insemination. The results showed that the day of insemination and the three weeks before it were the most sensitive periods. Even mild heat during that time reduced the chance of successful pregnancy. Some effects were also seen after insemination when the heat was more extreme, while other time frames were not affected. This study also found that some cows may cope better with heat than others, suggesting that breeding cows for heat resistance could improve fertility. These findings can help farmers make better decisions about cow care during hot weather and guide future breeding programmes to improve success rates, especially as climate change leads to hotter conditions.

## 1. Introduction

Climate change, along with its associated heat stress, is an undeniable and persistent challenge that continues to impact various ecosystems. As global temperatures rise and extreme temperature fluctuations become more frequent in temperate climates, heat stress has emerged as a significant and growing challenge in farming, particularly affecting dairy cows [1,2]. Since 1975, the mean world temperature has increased by 0.15–0.20 °C per decade, and the current climatic models forecast a potential elevation in Earth’s mean temperature ranging from 3.3 °C to 5.7 °C by the end of the current century [3]. 

Heat stress is defined as the combined effects of internal and external factors that raise an animal’s core body temperature higher than it can dissipate, resulting in the inability to maintain thermal equilibrium [4]. Dairy production is highly vulnerable to climatic fluctuations due to its central importance in the animal industry. Heat stress, which accounts for over 63.9% of total national losses in this sector, leads to annual economic losses of approximately 1.5–1.7 billion USD in the United States [5,6,7]. By the end of the century, US prediction models project financial losses of 2.2 billion USD [8]. This rise in temperature not only negatively impacts profitability but also animal welfare and weakens immune systems by decreasing lymphocyte proliferation, neutrophil oxidative burst and phagocytosis [9,10]. Furthermore, it negatively affects productivity as it can cause a decline in milk quality, reducing the levels of lactose, protein, and fat, including beta-casein, as well as leading to a lower milk quantity [11,12,13]. In addition, heat stress alters metabolism and blood parameters by increasing insulin and cortisol levels, among other hormones and enzymes, which further impair immune function [14,15]. Furthermore, heat stress affects cow behaviour, as cows reduce dry matter intake and ruminating time to cope with high temperatures, since rumination generates internal heat [11,16]. Additionally, it leads to reduced lying time and increased standing time, allowing cows to maximise their contact with the air to cool their bodies [17]. 

Beyond its impact on milk production and immunity, heat stress also significantly reduces fertility [18]. Moreover, as global warming intensifies and genetic selection for high-yielding cows continues, reproductive decline is projected to become a critical constraint for dairy farming [19]. Heat stress disrupts key reproductive processes, including follicular growth, hormonal balance, and embryonic development [20,21,22]. Specifically, elevated ambient temperatures reduce oestrus duration and intensity [23] while simultaneously altering reproductive hormones such as follicle-stimulating hormone (FSH), luteinizing hormone (LH), and oestradiol [19,24]. As a result, these hormonal shifts impair follicular dominance and development, leading to skewed follicle size distribution, disrupted oocyte competence, and reduced inhibin levels. Furthermore, elevated preovulatory FSH concentrations during hotter seasons exacerbate reproductive inefficiency [24,25,26]. Consequently, these disruptions contribute to prolonged intervals to first calving and extended calving intervals, further hindering reproductive performance in dairy cattle [18,27]. In addition, the adverse effects of heat stress extend to luteolytic mechanisms and uterine function, as it modulates prostaglandin F2α (PGF2α) secretion in endometrial tissues [28]. This hormonal imbalance also compromises early embryonic survival by reducing blastocyst rates and causing cytoplasmic changes [29,30]. 

Moreover, studies have consistently reported lower conception rates under thermal stress, highlighting the detrimental impact of heat stress on overall reproductive success [23,31]. While heat exposure on the day of service may significantly influence conception rates [23], services conducted during hot seasons are typically preceded and followed by elevated temperatures. The impact of these surrounding days may be as significant as, or even more critical than, the conditions on the day of service. A high ambient temperature during the service period has been consistently linked to a reduced conception rate [32,33,34]. However, the specific effects of heat exposure at particular times remain unclear. Accordingly, this study aimed to investigate the effect of heat stress during different periods before and after insemination to identify the most critical period affecting service success in dairy cows.

## 2. Materials and Methods

### 2.1. Animals

This research was carried out at a single farm situated in the southern region of Hungary in a continental climate (coordinates: 46.39, 20.26). This study analysed 47,199 insemination events from 2002 to 2024 among 6751 Holstein-Freisen cows who originated from 4121 dams and 329 sires. The average parity in our study was 1.61 ± 1.27, with an average number of inseminations per cow of 2.88 ± 1.36. This farm was chosen as they had all the necessary accurate records concerning the insemination parameters and because this farm received monthly veterinary reproductive monitoring visits. The study periods were selected based on biological principles. The long pre-insemination period, spanning from 21 days to 6 days before insemination (P1), corresponds to the growth and development of ovarian follicles [35,36,37]. Next, a short pre-insemination heat stress period from 5 days to 2 days before insemination (P2) was considered, as it is characterised by significant hormonal changes that influence reproductive processes and the final growth of the dominant follicle [38,39,40]. The ovulation preparation and peri-insemination period from 1 day before to 1 day after insemination (P3) encompasses ovulation, sperm capacitation, sperm survival in the female reproductive tract, and fertilisation [41,42,43]. Following this, the early post-insemination heat stress period from 2 days to 7 days after insemination (P4) was examined, as it covers crucial early developmental stages, including zygote cleavage and embryo migration to the uterus [44,45,46]. Finally, the long post-insemination period from 8 days to 30 days after insemination (P5) was included, as it is associated with embryo development, blastocyst growth, maternal recognition of pregnancy, and the initiation of implantation [47,48,49,50,51].

### 2.2. Conception

Only the artificial insemination data were used in the study and success was defined as a binary outcome (1 = pregnancy, 0 = no pregnancy). All the inseminations were performed 12 h after detecting signs of oestrus. The cows underwent manual rectal palpation around 40–45 days post-service during the next monthly herd visit by the veterinarian if no further signs of oestrus were observed. Services were considered unsuccessful if a negative pregnancy diagnosis was made between 40 and 100 days post-service, or if at least one subsequent oestrus was documented within 100 days after service.

### 2.3. Meteorological Data

Temperature and humidity were collected on a daily basis at a meteorological station belonging to the Hungarian Meteorological Service, located 13 km from the farm. The calculation of a temperature-humidity index was performed using the method reported by Dikmen et al. [52]:

THI=(1.8×T+32)−(0.55−0.55×RH/100)×(1.8×T−26)

where:

T = air temperature (°C)
RH = relative humidity (%)

### 2.4. Statistical Analysis

Statistical analyses were performed in R (version 4.4.1; R Core [53]). First, the effect of the Temperature–Humidity Index (THI) on the day of insemination was evaluated using a GLMM with a binomial distribution and logit link, fitted via the lme4 package [54]. The THI was categorized into thresholds (<45, 45, 46, …, ≥77) with THI ≤ 44 as the reference, and random intercepts were included for parity, year, semen, cow and inseminator to account for clustering. Odds ratios (ORs) with 95% confidence intervals (CIs) were calculated.

$$logit(P\left(Success\right))=\beta_{0}+\beta \cdot THI\_threshold+\sum_{m=1}^{5}u_{m}$$

where:

logit(P(Success)) = log odds of the probability of insemination success
$\beta_{0}$ = intercept
β⋅THI_threshold = the fixed effect of the THI threshold
$\sum_{m=1}^{5}u_{m}$ = the sum of the random intercepts (parity, year, semen, cow and inseminator)

Second, the average THI per period P1, P2, P3, P4, and P5 was categorized into four levels based on the observed relationship between the mean THI on the day of breeding and the resulting insemination success, which is similar to previous research describing thresholds of 70 [55] and 72 [56], respectively. We defined no heat stress as THI < 60, small heat stress as 60 ≤ THI < 68, medium heat stress as 68 ≤ THI < 72, and severe heat stress as THI ≥ 72, with no heat stress as the reference. A GLMM was fitted with these categories as fixed effects and random intercepts for cow, year, semen, parity and inseminator. Odds ratios (ORs) with 95% confidence intervals (CIs) were calculated.

$$\mathrm{logit}(P(\mathrm{Success}))=\beta_{0}+\sum_{p=1}^{5}\sum_{k=1}^{3}\beta_{p,k}\cdot I\left({\mathrm{HeatStress}}_{p}=k\right)+\sum_{m=1}^{5}u_{m}$$

where:

logit(P(Success)) = log odds of the probability of insemination success
$\beta_{0}\;$= the intercept, representing the log odds of success under the reference category, which is No Heat Stress (THI < 60).
p = the period (P1, P2, P3, P4, or P5).
k = the heat stress category (1: Small, 2: Medium, 3: Severe).
$\beta_{p,k}$ = the coefficient for the heat stress category *k* in period *p*.
$I\left({\mathrm{HeatStress}}_{p}=k\right)$ = an indicator variable that is 1 if the cow is in heat stress level *k* during period *p*, and 0 otherwise.
$\sum_{m=1}^{5}u_{m}$ = the sum of random intercepts (parity, year, semen, cow and inseminator)

Third, for periods showing significant heat stress effects (P1, P2, and P3), heritability was estimated using a Bayesian animal model in the MCMCglmm package [57]. A probit threshold model was used to estimate heritability on the liability scale, assuming an underlying continuous liability for the binary insemination success trait. The model included fixed effects for heat stress levels and random genetic effects modelled independently for each level within P1, P2, and P3. Pedigree data were used to construct an inverse additive genetic relationship matrix (A^−1^) via the pedigreemm package [58]. Heritability $(h^{2})\;$ was calculated as follows:

$$h^{2}=\frac{{\sigma^{2}}_{a}}{\left({\sigma^{2}}_{a}+\sigma^{2}\_Env+R\right)}$$

where:

$h^{2}$ = Heritability: proportion of total phenotypic variance attributable to additive genetic variance.
${\sigma^{2}}_{a}$ = Additive genetic variance: variance in insemination success due to genetic differences among cows.
$\sigma^{2}\_Env$ = variance due to year, semen, inseminator, and parity.
R = residual variance, fixed at 1 for binary outcomes on the liability scale.

## 3. Results

The THI on the day of insemination had a significant (*p* < 0.001) effect on insemination success in dairy cows. At lower THI values (≤50), there was no significant (*p* > 0.05) effect on the probability of insemination success, as indicated by odds ratios (OR) close to one. However, as the THI increased beyond this threshold, a gradual decline in success rates was observed (Table 1). A significant decrease in insemination success began at THI = 59 (B = −0.14, OR = 0.87, *p* = 0.03), suggesting a significant reduction of 13% in the odds of success compared to the reference category (THI ≤ 44). The decline became more pronounced at a THI = 60 (B = −0.18, OR = 0.84, *p* = 0.007), indicating a further reduction in success probability. A critical threshold appeared around THI = 62–64, where the odds of success dropped significantly (THI = 62: OR = 0.73, *p* < 0.001; THI 64: OR = 0.71, *p* < 0.001). Beyond THI = 68, the effect became even more severe, with OR values consistently below 0.65, indicating a significant reduction of more than 35% in insemination success compared to lower THI levels. At THI = 74, the odds of insemination success were reduced significantly by 57% (B = −0.85, OR = 0.43, *p* < 0.001). The most severe impact was observed at THI = 76 (B = −1.21, OR = 0.30, *p* < 0.001), where insemination success was reduced by 70% compared to the reference class.

The impact of heat stress on insemination success was analysed across five distinct time periods relative to insemination (P1, P2, P3, P4, and P5). Based on previous results, the cows were categorised into four heat stress levels: no heat stress, small heat stress, medium heat stress, and severe heat stress. Heat stress experienced during P1 had a significant (*p* < 0.001) negative impact on the odds of successful insemination. Cows exposed to small, medium, and severe heat stress had significantly lower odds of success compared to those under no heat stress conditions, with medium heat stress showing the most substantial effect—a 30% reduction in the odds of success (Table 2). The odds ratios for the three heat stress levels were as follows: small heat stress (OR = 0.823, 95% CI: 0.759–0.891), medium heat stress (OR = 0.697, 95% CI: 0.625–0.777), and severe heat stress (OR = 0.773, 95% CI: 0.675–0.885). In contrast, heat stress in P2 did not have a significant effect on insemination success (*p* > 0.05), with no statistically significant differences observed among the small, medium, and severe heat stress categories. During the peri-insemination period P3, small and medium heat stress did not significantly (*p* > 0.05) affect the odds of success compared to no heat stress conditions. However severe heat stress was the only category to show a significant (*p* < 0.001) negative effect. Cows under severe heat stress had significantly lower odds of successful insemination compared to those without heat stress (OR = 0.693, 95% CI: 0.565–0.850). In the short period after insemination (P4), heat stress did not show any significant (*p* > 0.05) effects on insemination success. The odds ratios for all levels of heat stress were close to one. In P5, severe heat stress again showed a significant negative effect on insemination success. Cows under severe heat stress had a 15% reduction in the odds of successful insemination (OR = 0.854, 95% CI: 0.743–0.981). However, no significant effects were found for small and medium heat stress in this period.

The heritability of the insemination results on the liability scale increased with the severity of heat stress during period P1, rising from 0.048 under no heat stress conditions (THI < 60) to 0.129 under severe heat stress (THI ≥ 72). Similar trends were observed for periods P3 and P5, with heritability increasing from 0.048 and 0.040, respectively, under no heat stress conditions to 0.124 and 0.140, respectively, under severe heat stress (Table 3).

## 4. Discussion

While the detrimental effects of heat stress on reproduction are well documented, the influence of its timing relative to insemination remains insufficiently understood. Specifically, whether heat stress occurring long before, shortly before, around, shortly after, or long after insemination has distinct impacts on success rates has not been thoroughly investigated. Identifying these critical windows could enable dairy producers to implement targeted interventions, such as enhanced cooling systems or adjusted breeding protocols, to mitigate the effects of heat stress and optimise reproductive outcomes. 

The present findings show that insemination results are significantly affected by THI levels on the day of insemination, with a critical threshold identified at THI = 60. Similarly, Ojo et al. [18] and Kipp et al. [59] found that a THI above 60 around the time of insemination negatively affects conception rates and prolongs the interval from the first to the last insemination. These findings may be explained, in part, by the fact that heat stress impairs the uterine environment by increasing uterine temperature, which might affect semen quality [60]. According to Llamas-Luceño et al. [61], a THI exceeding 60 correlates with reduced semen volume, fewer viable doses, and lower sperm concentration and motility. On the other hand, the impact may also be related to oestrus behaviour, as cows under heat stress often exhibit shorter or less detectable oestrus phases and hormonal imbalances, resulting in poorly timed inseminations when the THI exceeds 60 [62].

The present findings showed that heat stress significantly reduced insemination success during the P1 period across all stress levels between 17% and 31% and during the P3 and P5 periods under severe heat stress by 31% and 15%, respectively. In contrast, no significant effects were observed in the P2 and P4 periods, regardless of heat stress intensity. These patterns suggest that the timing and severity of heat stress are critical determinants of reproductive success.

The period P1 appears to be the most susceptible to heat stress, as even small levels of heat stress can significantly affect insemination outcomes compared to other timeframes. Our findings are consistent with [56,63,64,65], who reported a marked decrease in conception rates when heat stress occurred during the early stages of the oestrous cycle. This vulnerability may be explained by the fact that the 21 day to 6 day window before insemination coincides with follicular development and early oocyte maturation, critical phases in reproductive biology [37,66,67]. The consistent decline in insemination success across all levels of heat stress highlights the sensitivity of these reproductive processes. Heat stress is known to disrupt follicular growth by altering hormonal profiles, specifically by reducing levels of oestradiol and progesterone, which are essential for oocyte competence [63,68,69,70]. It also impairs granulosa cell function and induces oxidative stress, leading to apoptosis in follicular cells, increased follicular atresia, and diminished oocyte quality [71,72,73]. These compromised oocytes are less likely to be successfully fertilised, which explains the observed reduction in success odds.

The present findings indicate that the peri-ovulatory period is particularly vulnerable to severe heat stress when the Temperature–Humidity Index (THI) exceeds 72, resulting in a 31% reduction in insemination success compared to thermoneutral conditions. Our findings align with previous studies that reported a negative impact of the THI on conception rates around the day of insemination, with thresholds of 70 [55], 75 in Spain [33], 72 [56], and 73 [65]. This threshold effect suggests that while small to moderate heat stress may be buffered by physiological adaptations, such as increased respiration and improved uterine blood flow, severe stress overwhelms these mechanisms and disrupts key reproductive processes [74]. This may be explained by the fact that an elevated maternal body temperature impairs sperm motility and viability, causes oxidative damage to oocytes and zygotes, and alters the composition of oviductal fluid, thereby hindering gamete interactions [20]. Additionally, acute heat stress during the peri-ovulatory period adversely affects hormonal profiles, particularly by suppressing luteinising hormone (LH) surges, which are essential for ovulation, oocyte maturation, and corpus luteum formation [75,76,77]. This hormonal disruption underscores the sensitivity of the ovulatory process, which resembles an inflammatory response regulated by intrafollicular proteins and cytokines [41]. Elevated intraovarian temperatures further compromise ovulation and oocyte quality, leading to degeneration and reduced fertilisation potential [78,79,80]. Sperm are also directly affected; even brief exposure to heat shock significantly decreases their viability, motility, and fertilising capacity [81,82]. Moreover, heat stress during fertilisation contributes to oxidative stress and disrupts mechanisms that prevent polyspermy, thereby reducing the developmental competence of the resulting zygotes [83]. While preimplantation embryos may exhibit some resilience depending on their developmental stage, early cleavage stages, such as the two-cell embryo, remain particularly susceptible to heat-induced damage, further contributing to fertility decline under a high thermal load [84].

Heat stress during the P5 period significantly reduced insemination success by 15%, whereas moderate and mild heat stress had no noticeable effect. These results are consistent with those reported by [32,65,85]. According to López-Gatius [86], the highest risk of pregnancy loss due to heat stress occurs during the early embryonic phase, specifically between 8 and 17 days of gestation. This vulnerability may be explained by the critical roles of progesterone and interferon-tau in the establishment and maintenance of pregnancy in ruminants [87]. Heat-stressed embryos tend to develop more slowly, resulting in underdevelopment by days 16–17, a key stage in embryonic development [88,89]. Insufficient development leads to reduced production of interferon-tau, the hormone responsible for suppressing prostaglandin F2 alpha secretion. Without adequate interferon-tau, prostaglandin release induces regression of the corpus luteum (CL), jeopardizing pregnancy maintenance [90]. In addition, heat stress likely disrupts the uterine environment through mechanisms such as reduced blood flow or altered progesterone levels, further impairing implantation [20,91,92]. Direct thermal damage to embryos during the peri-implantation phase may also contribute to increased embryonic mortality [93]. Overall, the findings suggest that while livestock may tolerate moderate heat stress without adverse effects on pregnancy, severe heat stress exceeds their adaptive capacity, leading to pregnancy loss. This highlights the critical need for effective heat management strategies during the post-insemination period.

Heat stress during the period from 5 days to 2 days before insemination did not significantly affect insemination success (*p* > 0.05), regardless of the heat stress level. This lack of effect may be attributed to the oocyte beginning to produce heat shock protein 70 (HSP70), which offers cellular protection, during this period [94,95]. Additionally, this window coincides with final follicular maturation and dominant follicle selection, a stage that may be less sensitive to heat stress due to intrinsic antioxidant defences [96]. It also overlaps with a peak in oestrogen levels, which has been shown to enhance the expression of heat shock cognate 70 and HSP90 in rats [97].

In the present study, no significant effect of heat stress was observed during the P4 period (*p* > 0.05). Previous research is in line with our findings [84,98,99], as they found that morulae and blastocysts are examples of advanced embryos that have developed thermotolerance. This apparent resilience may be explained by findings from Sakatani et al. [100], who reported increased expression of the HSPA14 gene, a member of the HSP70 family, in morula-stage embryos exposed to thermal stress (40 °C for 24 h). HSPA14 is considered a major inducible gene in the heat shock response and plays a protective role against embryonic mortality [101]. Additionally, Sakatani et al. [102] found that heat stress had minimal impact on antioxidant gene expression during the morula stage, as it did not alter reactive oxygen species (ROS) production in embryos on days 4 and 6. However, levels of the antioxidant glutathione were found to increase at later embryonic stages [103].

The present findings indicate a pronounced increase in the heritability of insemination success on the liability scale with escalating heat stress severity, particularly during the P1, P2, and P3 periods. As heat stress intensified from no stress (THI < 60) to severe stress (THI > 72), heritability estimates increased from 0.048 to 0.129 in the P5 period, from 0.048 to 0.124 in the P3 period, and from 0.040 to 0.140 in the P1 period. These findings align with those of [18,104], who reported that the heritability of conception rate increased with rising heat stress levels. Analysing insemination success as a binary fertility trait on the liability scale using a probit threshold model [105] revealed that heritability values reflect the proportion of variance in liability attributable to additive genetic effects. The heritability estimates in this study are higher than those reported in previous studies [18,106,107]. This discrepancy may be attributed to the use of a probit function to calculate heritability on the liability scale, which tends to yield higher estimates compared to linear models [108,109]. Toghiani et al. [110], who also employed the liability scale, reported heritability estimates for pregnancy status ranging from 0.09 to 0.15 using univariate and multivariate analyses. The observed increase in heritability under severe heat stress suggests that genetic differences become more pronounced under challenging conditions, allowing favourable genotypes with enhanced heat tolerance or fertility resilience to more readily exceed the liability threshold for reproductive success [109]. Conversely, the relatively low heritability under no heat stress (0.040–0.048) underscores the predominance of environmental factors in optimal conditions, as noted by Pryce et al. [111]. However, the higher heritability observed under severe stress (0.12–0.14) indicates a greater potential for genetic selection in increasingly prevalent heat-stressed environments, aligning with climate change projections.

## 5. Conclusions

The present study demonstrated that heat stress negatively affects insemination success in dairy cows, with a THI threshold of 60 indicating the onset of adverse effects on the day of insemination. The most critical period was 21 days to 6 days before insemination, while the peri-insemination (1 day before to 1 day after) and post-insemination (8 days to 30 days after) periods were also susceptible to high heat stress. In contrast, the periods from 5 days to 2 days before and 2 days to 7 days after insemination showed no significant impact of heat stress on insemination success. Implementing cooling strategies during these vulnerable periods may help mitigate heat stress effects. Additionally, genetic variability in response to heat stress was more pronounced under severe conditions, indicating potential for breeding heat-resilient animals. Future prospective studies should combine continuous core body temperature monitoring, such as the rectal temperature, with THI assessment and hormone profiling during critical reproductive windows. This would allow direct quantification of individual thermoregulatory responses and their relationship with fertility outcomes under varying environmental conditions.

## Figures and Tables

**Table 1:** Odds ratio of insemination success over the THI threshold on the day of insemination.

THI Threshold	B Slope	Odds Ratio	95% Confidence Interval	*N* ≥ Threshold	*p*	Success Rate (%)
≤44	Reference					
45	0.02	1.02	(0.89–1.18)	1053	0.75	34.9
46	0	1	(0.88–1.15)	1110	0.952	34
47	0.02	1.02	(0.9–1.15)	1377	0.761	33.8
48	0.08	1.09	(0.94–1.26)	912	0.282	34.6
49	0.03	1.03	(0.9–1.19)	1067	0.637	32.6
50	0.01	1.02	(0.88–1.17)	1119	0.832	32.8
51	−0.07	0.93	(0.81–1.07)	1132	0.326	31
52	0.06	1.06	(0.92–1.23)	970	0.435	33.3
53	−0.09	0.91	(0.79–1.05)	1047	0.2	32.7
54	−0.06	0.94	(0.81–1.09)	1018	0.386	29.8
55	−0.1	0.91	(0.79–1.05)	1121	0.181	31.6
56	−0.05	0.95	(0.82–1.09)	1118	0.458	30.9
57	−0.12	0.89	(0.77–1.02)	1170	0.089	29.9
58	−0.03	0.98	(0.84–1.14)	903	0.75	31.3
59	−0.14	0.87	(0.76–0.99)	1315	0.03 *	29.8
60	−0.18	0.84	(0.73–0.95)	1346	0.007 *	28
61	−0.11	0.9	(0.78–1.04)	1057	0.145	29
62	−0.31	0.73	(0.64–0.84)	1252	<0.001 *	26
63	−0.12	0.88	(0.77–1.02)	1108	0.083	30.4
64	−0.34	0.71	(0.61–0.82)	1156	<0.001 *	26
65	−0.29	0.75	(0.65–0.87)	1053	<0.001 *	26.6
66	−0.44	0.65	(0.56–0.75)	1143	<0.001 *	23.8
67	−0.45	0.64	(0.55–0.74)	1149	<0.001 *	23.6
68	−0.42	0.65	(0.58–0.74)	1541	<0.001 *	24.3
69	−0.52	0.59	(0.51–0.69)	1205	<0.001 *	22.5
70	−0.56	0.57	(0.5–0.66)	1261	<0.001 *	23.5
71	−0.53	0.59	(0.51–0.69)	1082	<0.001 *	22.7
72	−0.63	0.53	(0.45–0.62)	1080	<0.001 *	20.3
73	−0.64	0.53	(0.44–0.63)	878	<0.001 *	21.5
74	−0.85	0.43	(0.36–0.51)	844	<0.001 *	19.7
75	−0.64	0.53	(0.44–0.63)	765	<0.001 *	21.8
76	−1.21	0.3	(0.23–0.39)	463	<0.001 *	14.7
77	−0.79	0.46	(0.37–0.55)	697	<0.001 *	20.9

* Significant (*p* < 0.05).

**Table 2:** Odds ratios for insemination success under different levels of heat stress across periods around service.

Time Period	Heat Stress Category	Odds Ratio	95% CI (Odds Ratio)	*N*	*p*-Value
P1	No HS	Reference		29,028	
	Small Heat Stress	0.823	(0.759, 0.891)	8381	<0.001 ***
	Medium Heat Stress	0.697	(0.625, 0.777)	6339	<0.001 ***
	Severe Heat Stress	0.773	(0.675, 0.885)	3451	<0.001 ***
P2	No HS	Reference		28,924	
	Small Heat Stress	0.973	(0.886, 1.069)	9343	0.574
	Medium Heat Stress	0.913	(0.792, 1.053)	5369	0.212
	Severe Heat Stress	1.005	(0.844, 1.197)	3536	0.952
P3	No HS	Reference		28,892	
	Small Heat Stress	0.926	(0.835, 1.027)	9343	0.147
	Medium Heat Stress	0.887	(0.757, 1.039)	5396	0.137
	Severe Heat Stress	0.693	(0.565, 0.850)	3536	<0.001 ***
P4	No HS	Reference		29,028	
	Small Heat Stress	0.941	(0.858, 1.032)	9247	0.195
	Medium Heat Stress	0.935	(0.814, 1.075)	5318	0.345
	Severe Heat Stress	0.922	(0.776, 1.096)	3606	0.353
P5	No HS	Reference		29,827	
	Small Heat Stress	1.058	(0.978, 1.145)	8600	0.165
	Medium Heat Stress	0.941	(0.840, 1.054)	5815	0.300
	Severe Heat Stress	0.854	(0.743, 0.981)	2957	0.026 *

* Significant (*p* < 0.05), *** Significant (*p* < 0.01).

**Table 3:** Liability scale heritability estimates of insemination success across different periods and levels of heat stress.

Period	Heat Stress Category	Heritability	CrI_2.5	CrI_97.5
P1	No Heat Stress	0.048	0.022	0.100
	Small Heat Stress	0.081	0.033	0.173
	Medium Heat Stress	0.112	0.044	0.241
	Severe Heat Stress	0.129	0.047	0.275
P3	No Heat Stress	0.048	0.022	0.099
	Small Heat Stress	0.079	0.032	0.17
	Medium Heat Stress	0.107	0.039	0.231
	Severe Heat Stress	0.124	0.043	0.265
P5	No Heat Stress	0.040	0.020	0.095
	Small Heat Stress	0.082	0.031	0.176
	Medium Heat Stress	0.093	0.035	0.197
	Severe Heat Stress	0.140	0.047	0.295

## Data Availability

The data presented in this study are available on request from the corresponding author. The data are not publicly available due to the fact that it was obtained from dairy farmers and we do not have their consent for publication.

## References

[1] Georgiades P., Economou T., Proestos Y., Araya J., Lelieveld J., Neira M. (2025). Global Projections of Heat Stress at High Temporal Resolution Using Machine Learning. Earth Syst. Sci. Data.

[2] Wankar A.K., Rindhe S.N., Doijad N.S. (2021). Heat Stress in Dairy Animals and Current Milk Production Trends, Economics, and Future Perspectives: The Global Scenario. Trop. Anim. Health Prod..

[3] Lee H., Calvin K., Dasgupta D., Krinner G., Mukherji A., Thorne P., Trisos C., Romero J., Aldunce P., Barret K., Lee H., Romero J., Core Writing Team (2023). IPCC, 2023: Climate Change 2023: Synthesis Report, Summary for Policymakers. Contribution of Working Groups I, II and III to the Sixth Assessment Report of the Intergovernmental Panel on Climate Change.

[4] Yousef M.K. (1985). Stress Physiology in Livestock. Volume I. Basic Principles.

[5] North M.A., Franke J.A., Ouweneel B., Trisos C.H. (2023). Global Risk of Heat Stress to Cattle from Climate Change. Environ. Res. Lett..

[6] St-Pierre N.R., Cobanov B., Schnitkey G. (2003). Economic Losses from Heat Stress by US Livestock Industries1. J. Dairy Sci..

[7] Cartwright S.L., Schmied J., Karrow N., Mallard B.A. (2023). Impact of Heat Stress on Dairy Cattle and Selection Strategies for Thermotolerance: A Review. Front. Vet. Sci..

[8] Mauger G., Bauman Y., Nennich T., Salathé E. (2015). Impacts of Climate Change on Milk Production in the United States. Prof. Geogr..

[9] Gupta S., Sharma A., Joy A., Dunshea F.R., Chauhan S.S. (2022). The Impact of Heat Stress on Immune Status of Dairy Cattle and Strategies to Ameliorate the Negative Effects. Animals.

[10] Do Amaral B.C., Connor E.E., Tao S., Hayen J., Bubolz J., Dahl G.E. (2010). Heat Stress Abatement during the Dry Period Influences Prolactin Signaling in Lymphocytes. Domest. Anim. Endocrinol..

[11] Chen L., Thorup V.M., Kudahl A.B., Østergaard S. (2024). Effects of Heat Stress on Feed Intake, Milk Yield, Milk Composition, and Feed Efficiency in Dairy Cows: A Meta-Analysis. J. Dairy Sci..

[12] Cowley F.C., Barber D.G., Houlihan A.V., Poppi D.P. (2015). Immediate and Residual Effects of Heat Stress and Restricted Intake on Milk Protein and Casein Composition and Energy Metabolism. J. Dairy Sci..

[13] Dunn R.J.H., Mead N.E., Willett K.M., Parker D.E. (2014). Analysis of Heat Stress in UK Dairy Cattle and Impact on Milk Yields. Environ. Res. Lett..

[14] Ataallahi M., Cheon S.N., Park G.-W., Nugrahaeningtyas E., Jeon J.H., Park K.-H. (2023). Assessment of Stress Levels in Lactating Cattle: Analyzing Cortisol Residues in Commercial Milk Products in Relation to the Temperature-Humidity Index. Animals.

[15] Zachut M., Kra G., Nemes-Navon N., Ben-Aharon N., Moallem U., Lavon Y., Jacoby S. (2020). Seasonal Heat Load Is More Potent than the Degree of Body Weight Loss in Dysregulating Immune Function by Reducing White Blood Cell Populations and Increasing Inflammation in Holstein Dairy Cows. J. Dairy Sci..

[16] Antanaitis R., Džermeikaitė K., Krištolaitytė J., Juodžentytė R., Stankevičius R., Palubinskas G., Rutkauskas A. (2024). Short-Term Effects of Heat Stress on Cow Behavior, Registered by Innovative Technologies and Blood Gas Parameters. Animals.

[17] Ninomiya S., Goto Y., Onishi H., Kurachi M., Ito A. (2023). Lying Posture as a Behavioural Indicator of Heat Stress in Dairy Cows. Appl. Anim. Behav. Sci..

[18] Ojo T.O., Vandenplas J., Mulder H.A., van Pelt M.L., Calus M.P.L. (2025). Genetic Analysis of the Impact of Heat Stress on Fertility Traits in Dairy Cows in the Netherlands. J. Dairy Sci..

[19] Roth Z. (2017). Effect of Heat Stress on Reproduction in Dairy Cows: Insights into the Cellular and Molecular Responses of the Oocyte. Annu. Rev. Anim. Biosci..

[20] Hansen P.J. (2009). Effects of Heat Stress on Mammalian Reproduction. Philos. Trans. R. Soc. B Biol. Sci..

[21] Silva C.F., Sartorelli E.S., Castilho A.C.S., Satrapa R.A., Puelker R.Z., Razza E.M., Ticianelli J.S., Eduardo H.P., Loureiro B., Barros C.M. (2013). Effects of Heat Stress on Development, Quality and Survival of Bos Indicus and Bos Taurus Embryos Produced in Vitro. Theriogenology.

[22] Sigdel A., Liu L., Abdollahi-Arpanahi R., Aguilar I., Peñagaricano F. (2020). Genetic Dissection of Reproductive Performance of Dairy Cows under Heat Stress. Anim. Genet..

[23] Schüller L.K., Michaelis I., Heuwieser W. (2017). Impact of Heat Stress on Estrus Expression and Follicle Size in Estrus under Field Conditions in Dairy Cows. Theriogenology.

[24] Wolfenson D., Roth Z. (2019). Impact of Heat Stress on Cow Reproduction and Fertility. Anim. Front..

[25] Aggarwal A., Upadhyay R. (2013). Heat Stress and Animal Productivity.

[26] Ingraham R.H., Gillette D.D., Wagner W.D. (1974). Relationship of Temperature and Humidity to Conception Rate of Holstein Cows in Subtropical Climate. J. Dairy Sci..

[27] Stefanska B., Pruszynska-Oszmalek E., Fievez V., Purwin C., Nowak W. (2024). Impact of Heat Stress during Close-up Dry Period on Performance, Fertility and Immunometabolic Blood Indices of Dairy Cows: Prospective Cohort Study. Sci. Rep..

[28] Putney D.J., Gross T.S., Thatcher W.W. (1988). Prostaglandin Secretion by Endometrium of Pregnant and Cyclic Cattle at Day 17 after Oestrus in Response to In-Vitro Heat Stress. Reproduction.

[29] Park S.Y., Kim E.Y., JeOn K., Cui X., Lee W.D., Kim N., Park S.P., Lim J.H. (2007). Survivin Acts as Anti-apoptotic Factor during the Development of Bovine Pre-implantation Embryos. Mol. Reprod. Dev..

[30] Da Costa R.A., dos Santos M.L.P., Castro I.C., Mattiello M., Souza J.A., Almeida W.F., Boettcher D.L., Cuchi D., Paim B.H.R., Vieira F.M.C. (2021). 126 Effect of Astaxanthin Supplementation on in Vitro Embryo Development and Immune Response of Dairy Cows. Reprod. Fertil. Dev..

[31] Nabenishi H., Ohta H., Nishimoto T., Morita T., Ashizawa K., Tsuzuki Y. (2011). Effect of the Temperature-Humidity Index on Body Temperature and Conception Rate of Lactating Dairy Cows in Southwestern Japan. J. Reprod. Dev..

[32] Jordan E.R. (2003). Effects of Heat Stress on Reproduction. J. Dairy Sci..

[33] García-Ispierto I., López-Gatius F., Bech-Sabat G., Santolaria P., Yániz J.L., Nogareda C., De Rensis F., López-Béjar M. (2007). Climate Factors Affecting Conception Rate of High Producing Dairy Cows in Northeastern Spain. Theriogenology.

[34] De Rensis F., Garcia-Ispierto I., López-Gatius F. (2015). Seasonal Heat Stress: Clinical Implications and Hormone Treatments for the Fertility of Dairy Cows. Theriogenology.

[35] Spicer L.J., Echternkamp S.E. (1986). Ovarian Follicular Growth, Function and Turnover in Cattle: A Review1. J. Anim. Sci..

[36] Ginther O.J., Kastelic J.P., Knopf L. (1989). Composition and Characteristics of Follicular Waves during the Bovine Estrous Cycle. Anim. Reprod. Sci..

[37] Hafez E.S.E., Hafez B. (2013). Reproduction in Farm Animals.

[38] Wiltbank M.C., Souza A.H., Carvalho P.D., Cunha A.P., Giordano J.O., Fricke P.M., Baez G.M., Diskin M.G. (2014). Physiological and Practical Effects of Progesterone on Reproduction in Dairy Cattle. Animal.

[39] Sartori R., Haughian J.M., Shaver R.D., Rosa G.J.M., Wiltbank M.C. (2004). Comparison of Ovarian Function and Circulating Steroids in Estrous Cycles of Holstein Heifers and Lactating Cows. J. Dairy Sci..

[40] Dieleman S.J., Bevers M.M., Van Tol H.T.M., Willemse A.H. (1986). Peripheral Plasma Concentrations of Oestradiol, Progesterone, Cortisol, LH and Prolactin during the Oestrous Cycle in the Cow, with Emphasis on the Peri-Oestrous Period. Anim. Reprod. Sci..

[41] De Rensis F., Dall’Olio E., Gnemmi G.M., Tummaruk P., Andrani M., Saleri R. (2024). Interval from Oestrus to Ovulation in Dairy Cows—A Key Factor for Insemination Time: A Review. Vet. Sci..

[42] Driancourt M.A., Thatcher W.W., Terqui M., Andrieu D. (1991). Dynamics of Ovarian Follicular Development in Cattle during the Estrous Cycle, Early Pregnancy and in Response to PMSG. Domest. Anim. Endocrinol..

[43] Parrish J.J., Susko-Parrish J.L., Handrow R.R., Sims M.M., First N.L. (1989). Capacitation of Bovine Spermatozoa by Oviduct Fluid. Biol. Reprod..

[44] Hansen P.J., Tríbulo P. (2019). Regulation of Present and Future Development by Maternal Regulatory Signals Acting on the Embryo during the Morula to Blastocyst Transition–Insights from the Cow. Biol. Reprod..

[45] Memili E., First N.L. (2000). Zygotic and Embryonic Gene Expression in Cow: A Review of Timing and Mechanisms of Early Gene Expression as Compared with Other Species. Zygote.

[46] Picha Y., Tibary A., Memon M., Kasimanickam R., Sumar J. (2013). Chronology of Early Embryonic Development and Embryo Uterine Migration in Alpacas. Theriogenology.

[47] Lemley C.O., Camacho L.E., Vonnahme K.A. (2021). Maternal Recognition and Physiology of Pregnancy. Bovine Reproduction.

[48] Bazer F.W., Ott T.L., Spencer T.E. (1998). Endocrinology of the Transition from Recurring Estrous Cycles to Establishment of Pregnancy in Subprimate Mammals. Endocrinology of Pregnancy.

[49] Degrelle S.A., Campion E., Cabau C., Piumi F., Reinaud P., Richard C., Renard J.-P., Hue I. (2005). Molecular Evidence for a Critical Period in Mural Trophoblast Development in Bovine Blastocysts. Dev. Biol..

[50] Sponchiado M., Gomes N.S., Fontes P.K., Martins T., Del Collado M., Pastore A.d.A., Pugliesi G., Nogueira M.F.G., Binelli M. (2017). Pre-Hatching Embryo-Dependent and-Independent Programming of Endometrial Function in Cattle. PLoS ONE.

[51] Lonergan P., Fair T., Forde N., Rizos D. (2016). Embryo Development in Dairy Cattle. Theriogenology.

[52] Dikmen S., Alava E., Pontes E., Fear J.M., Dikmen B.Y., Olson T.A., Hansen P.J. (2008). Differences in Thermoregulatory Ability between Slick-Haired and Wild-Type Lactating Holstein Cows in Response to Acute Heat Stress. J. Dairy Sci..

[53] R Core Team (2020). RA Language and Environment for Statistical Computing.

[54] Bates D., Mächler M., Bolker B., Walker S. (2015). Fitting Linear Mixed-Effects Models Using Lme4. J. Stat. Softw..

[55] Ravagnolo O., Misztal I., Hoogenboom G. (2000). Genetic Component of Heat Stress in Dairy Cattle, Development of Heat Index Function. J. Dairy Sci..

[56] Morton J.M., Tranter W.P., Mayer D.G., Jonsson N.N. (2007). Effects of Environmental Heat on Conception Rates in Lactating Dairy Cows: Critical Periods of Exposure. J. Dairy Sci..

[57] Hadfield J.D. (2010). MCMC Methods for Multi-Response Generalized Linear Mixed Models: The MCMCglmm R Package. J. Stat. Softw..

[58] Vazquez A.I., Bates D.M., Rosa G.J.M., Gianola D., Weigel K.A. (2010). Technical Note: An R Package for Fitting Generalized Linear Mixed Models in Animal Breeding1. J. Anim. Sci..

[59] Kipp C., Brügemann K., Yin T., Halli K., König S. (2021). Genotype by Heat Stress Interactions for Production and Functional Traits in Dairy Cows from an Across-Generation Perspective. J. Dairy Sci..

[60] Sammad A., Umer S., Shi R., Zhu H., Zhao X., Wang Y. (2020). Dairy Cow Reproduction under the Influence of Heat Stress. J. Anim. Physiol. Anim. Nutr. (Berl.).

[61] Llamas-Luceño N., Hostens M., Mullaart E., Broekhuijse M., Lonergan P., Van Soom A. (2020). High Temperature-Humidity Index Compromises Sperm Quality and Fertility of Holstein Bulls in Temperate Climates. J. Dairy Sci..

[62] Tippenhauer C.M., Plenio J.-L., Madureira A.M.L., Cerri R.L.A., Heuwieser W., Borchardt S. (2021). Timing of Artificial Insemination Using Fresh or Frozen Semen after Automated Activity Monitoring of Estrus in Lactating Dairy Cows. J. Dairy Sci..

[63] Wolfenson D., Roth Z., Meidan R. (2000). Impaired Reproduction in Heat-Stressed Cattle: Basic and Applied Aspects. Anim. Reprod. Sci..

[64] Schüller L.-K., Burfeind O., Heuwieser W. (2016). Effect of Short- and Long-Term Heat Stress on the Conception Risk of Dairy Cows under Natural Service and Artificial Insemination Breeding Programs. J. Dairy Sci..

[65] Schüller L.K., Burfeind O., Heuwieser W. (2014). Impact of Heat Stress on Conception Rate of Dairy Cows in the Moderate Climate Considering Different Temperature–Humidity Index Thresholds, Periods Relative to Breeding, and Heat Load Indices. Theriogenology.

[66] Adams G.P., Singh J. (2021). Ovarian Follicular and Luteal Dynamics in Cattle. Bovine Reproduction.

[67] Minela T., Gibb P., McBeth S., Santos A., Pursley J.R. (2023). Reduced Period from Follicular Wave Emergence to Luteolysis Generated Greater Steroidogenic Follicles and Estrus Intensity in Dairy Cows. Sci. Rep..

[68] De Rensis F., Saleri R., Garcia-Ispierto I., Scaramuzzi R., López-Gatius F. (2021). Effects of Heat Stress on Follicular Physiology in Dairy Cows. Animals.

[69] Boni R. (2019). Heat Stress, a Serious Threat to Reproductive Function in Animals and Humans. Mol. Reprod. Dev..

[70] Dovolou E., Giannoulis T., Nanas I., Amiridis G.S. (2023). Heat Stress: A Serious Disruptor of the Reproductive Physiology of Dairy Cows. Animals.

[71] Roth Z., Arav A., Bor A., Zeron Y., Braw-Tal R., Wolfenson D. (2001). Improvement of Quality of Oocytes Collected in the Autumn by Enhanced Removal of Impaired Follicles from Previously Heat-Stressed Cows. Reproduction.

[72] Wang Y., Yang C., Nahla Abdalla Hassan E., Li C., Yang F., Wang G., Li L. (2019). HO-1 Reduces Heat Stress-Induced Apoptosis in Bovine Granulosa Cells by Suppressing Oxidative Stress. Aging.

[73] Abdelnour S.A., Swelum A.A., Abd El-Hack M.E., Khafaga A.F., Taha A.E., Abdo M. (2020). Cellular and Functional Adaptation to Thermal Stress in Ovarian Granulosa Cells in Mammals. J. Therm. Biol..

[74] Negrón-Pérez V.M., Fausnacht D.W., Rhoads M.L. (2019). Invited Review: Management Strategies Capable of Improving the Reproductive Performance of Heat-Stressed Dairy Cattle. J. Dairy Sci..

[75] Roth Z. (2020). Influence of Heat Stress on Reproduction in Dairy Cows—Physiological and Practical Aspects. J. Anim. Sci..

[76] Al-Katanani Y.M., Paula-Lopes F.F., Hansen P.J. (2002). Effect of Season and Exposure to Heat Stress on Oocyte Competence in Holstein Cows1. J. Dairy Sci..

[77] Sartori R., Rosa G.J.M., Wiltbank M.C. (2002). Ovarian Structures and Circulating Steroids in Heifers and Lactating Cows in Summer and Lactating and Dry Cows in Winter. J. Dairy Sci..

[78] López-Gatius F., Hunter R.H.F. (2017). Clinical Relevance of Pre-Ovulatory Follicular Temperature in Heat-Stressed Lactating Dairy Cows. Reprod. Domest. Anim..

[79] Morita Y., Ozaki R., Mukaiyama A., Sasaki T., Tatebayashi R., Morishima A., Kitagawa Y., Suzumura R., Abe R., Tsukamura H. (2020). Establishment of Long-Term Chronic Recording Technique of in Vivo Ovarian Parenchymal Temperature in Japanese Black Cows. J. Reprod. Dev..

[80] Amaral C.S., Koch J., Correa Júnior E.E., Bertolin K., Mujica L.K.S., Fiorenza M.F., Rosa S.G., Nogueira C.W., Comim F.V., Portela V.V.M. (2020). Heat Stress on Oocyte or Zygote Compromises Embryo Development, Impairs Interferon Tau Production and Increases Reactive Oxygen Species and Oxidative Stress in Bovine Embryos Produced in Vitro. Mol. Reprod. Dev..

[81] Hendricks K.E.M., Martins L., Hansen P.J. (2009). Consequences for the Bovine Embryo of Being Derived from a Spermatozoon Subjected to Post-Ejaculatory Aging and Heat Shock: Development to the Blastocyst Stage and Sex Ratio. J. Reprod. Dev..

[82] Pérez-Crespo M., Pintado B., Gutiérrez-Adán A. (2008). Scrotal Heat Stress Effects on Sperm Viability, Sperm DNA Integrity, and the Offspring Sex Ratio in Mice. Mol. Reprod. Dev..

[83] Sakatani M., Yamanaka K., Balboula A.Z., Takenouchi N., Takahashi M. (2015). Heat Stress during in Vitro Fertilization Decreases Fertilization Success by Disrupting Anti-Polyspermy Systems of the Oocytes. Mol. Reprod. Dev..

[84] Hansen P.J. (2007). Exploitation of Genetic and Physiological Determinants of Embryonic Resistance to Elevated Temperature to Improve Embryonic Survival in Dairy Cattle during Heat Stress. Theriogenology.

[85] García-Ispierto I., López-Gatius F., Santolaria P., Yániz J.L., Nogareda C., López-Béjar M., De Rensis F. (2006). Relationship between Heat Stress during the Peri-Implantation Period and Early Fetal Loss in Dairy Cattle. Theriogenology.

[86] López-Gatius F. (2003). Is Fertility Declining in Dairy Cattle?: A Retrospective Study in Northeastern Spain. Theriogenology.

[87] Halloran K.M., Stenhouse C., Moses R.M., Seo H., Johnson G.A., Wu G., Bazer F.W. (2022). Progesterone and Interferon Tau Regulate Expression of Polyamine Enzymes during the Ovine Peri-Implantation Period†. Biol. Reprod..

[88] Kasimanickam R., Kasimanickam V. (2021). Impact of Heat Stress on Embryonic Development during First 16 Days of Gestation in Dairy Cows. Sci. Rep..

[89] Biggers B.G., Geisert R.D., Wetteman R.P., Buchanan D.S. (1987). Effect of Heat Stress on Early Embryonic Development in the Beef Cow. J. Anim. Sci..

[90] Sakai S., Yagi M., Fujime N., Kuse M., Sakumoto R., Yamamoto Y., Okuda K., Kimura K. (2021). Heat Stress Influences the Attenuation of Prostaglandin Synthesis by Interferon Tau in Bovine Endometrial Cells. Theriogenology.

[91] Samal L. (2013). Heat Stress in Dairy Cows-Reproductive Problems and Control Measures. Int. J. Livest. Res..

[92] Amaral C.d.S., Correa G.R.E., Serrano Mujica L.K., Fiorenza M.F., Rosa S.G., Nogueira C.W., Portela V.M., Comim F.V., Schoenau W., Smirnova N.P. (2021). Heat Stress Modulates Polymorphonuclear Cell Response in Early Pregnancy Cows: I. Interferon Pathway and Oxidative Stress. PLoS ONE.

[93] Ealy A.D., Drost M., Hansen P.J. (1993). Developmental Changes in Embryonic Resistance to Adverse Effects of Maternal Heat Stress in Cows1. J. Dairy Sci..

[94] Driancourt M.A., Guet P., Reynaud K., Chadli A., Catelli M.G. (1999). Presence of an Aromatase Inhibitor, Possibly Heat Shock Protein 90, in Dominant Follicles of Cattle. Reproduction.

[95] Edwards J.L., Hansen P.J. (1997). Differential Responses of Bovine Oocytes and Preimplantation Embryos to Heat Shock. Mol. Reprod. Dev. Inc. Gamete Res..

[96] Gupta S., Choi A., Yu H.Y., Czerniak S.M., Holick E.A., Paolella L.J., Agarwal A., Combelles C.M.H. (2011). Fluctuations in Total Antioxidant Capacity, Catalase Activity and Hydrogen Peroxide Levels of Follicular Fluid during Bovine Folliculogenesis. Reprod. Fertil. Dev..

[97] Krebs C.J., Jarvis E.D., Pfaff D.W. (1999). The 70-KDa Heat Shock Cognate Protein (Hsc73) Gene Is Enhanced by Ovarian Hormones in the Ventromedial Hypothalamus. Proc. Natl. Acad. Sci. USA.

[98] Sakatani M. (2017). Effects of Heat Stress on Bovine Preimplantation Embryos Produced in Vitro. J. Reprod. Dev..

[99] Bonilla A.Q.S., Oliveira L.J., Ozawa M., Newsom E.M., Lucy M.C., Hansen P.J. (2011). Developmental Changes in Thermoprotective Actions of Insulin-like Growth Factor-1 on the Preimplantation Bovine Embryo. Mol. Cell Endocrinol..

[100] Sakatani M., Alvarez N.V., Takahashi M., Hansen P.J. (2012). Consequences of Physiological Heat Shock Beginning at the Zygote Stage on Embryonic Development and Expression of Stress Response Genes in Cattle. J. Dairy Sci..

[101] Hassan F., Nawaz A., Rehman M.S., Ali M.A., Dilshad S.M.R., Yang C. (2019). Prospects of HSP70 as a Genetic Marker for Thermo-Tolerance and Immuno-Modulation in Animals under Climate Change Scenario. Anim. Nutr..

[102] Sakatani M., Kobayashi S.-I., Takahashi M. (2004). Effects of Heat Shock on in Vitro Development and Intracellular Oxidative State of Bovine Preimplantation Embryos. Mol. Reprod. Dev..

[103] Edwards J.L., King W.A., Kawarsky S.J., Ealy A.D. (2001). Responsiveness of Early Embryos to Environmental Insults: Potential Protective Roles of HSP70 and Glutathione. Theriogenology.

[104] Vinet A., Mattalia S., Vallée R., Bertrand C., Barbat A., Promp J., Cuyabano B.C.D., Boichard D. (2024). Effect of Temperature-Humidity Index on the Evolution of Trade-Offs between Fertility and Production in Dairy Cattle. Genet. Sel. Evol..

[105] Falconer D.S. (1996). Introduction to Quantitative Genetics.

[106] Berry D.P., Wall E., Pryce J.E. (2014). Genetics and Genomics of Reproductive Performance in Dairy and Beef Cattle. Animal.

[107] González-Recio O., Alenda R. (2005). Genetic Parameters for Female Fertility Traits and a Fertility Index in Spanish Dairy Cattle. J. Dairy Sci..

[108] Heringstad B., Rekaya R., Gianola D., Klemetsdal G., Weigel K.A. (2003). Genetic Change for Clinical Mastitis in Norwegian Cattle: A Threshold Model Analysis. J. Dairy Sci..

[109] Visscher P.M., Hill W.G., Wray N.R. (2008). Heritability in the Genomics Era—Concepts and Misconceptions. Nat. Rev. Genet..

[110] Toghiani S., Hay E., Sumreddee P., Geary T.W., Rekaya R., Roberts A.J. (2017). Genomic Prediction of Continuous and Binary Fertility Traits of Females in a Composite Beef Cattle Breed. J. Anim. Sci..

[111] Pryce J.E., Royal M.D., Garnsworthy P.C., Mao I.L. (2004). Fertility in the High-Producing Dairy Cow. Livest. Prod. Sci..

